# Pathopsychological characteristics of patients with extreme obesity 12 months after SADI-GP

**DOI:** 10.1192/j.eurpsy.2025.1392

**Published:** 2025-08-26

**Authors:** F. Császár, I. B. Bálint, R. Jávor, I. B. Bálint

**Affiliations:** 1 Psychiatry, Vas County Markusovszky University Teaching Hospital, Szombathely; 2 Department of Urology, Kanizsai Dorottya Hospital, Nagykanizsa; 3 Faculty of Humanities and Social Sciences, University of Pécs, Pécs, Hungary

## Abstract

**Introduction:**

Morbid obesity is correlated with hypertension, dyslipidaemia, prediabetes and T2DM. If lifestyle modifications and pharmacotherapeutic or mixed interventions fail, bariatric surgery is advised for morbidly obese patients. Psychological characteristics of such patients, especially after bariatric surgery, are a vastly under-researched area.

**Objectives:**

This research presents the short-term results of the LASAGNE trial (LAparoscopic Single-Anastomosis duodeno-ileal bypass with Gastric plication (SADI-GP) in the maNagEment of morbid obesity) regarding psychopathological values.

**Methods:**

This study used a cohort of consecutively admitted patients to assess complication rates and efficacy of SADI-GP. Patient recruitment began in October 2018 and ended in June 2019. Preoperative evaluation followed by surgery and postoperative follow-up visits (at 1, 3, 6 and 12 months) with Minnesota Multiphasic Personality Inventory (MMPI-2) recorded at 12-month follow-up. Participants’ age was between 18 and 65 years, with BMIs of > 40 (without comorbidity related to morbid obesity) or > 35 (with comorbidity related to morbid obesity, especially glucose metabolism). Psychological characteristics of patient groups were analyzed based on weight loss outcomes and Body Mass Index (BMI) changes. Substance Use Disorder was among other exclusion criteria.

**Results:**

Scales of affective function deficit (RCd, RC1, RC2, RC7) were elevated at 13 cases, scales of behavioural dysfunction (RC4, RC9) were elevated at 11 cases, scales of thought dysfunction (RC3, RC6, RC8) were elevated at 8 cases, RC2 and RC7 showed emotion dysregulation tendencies (Table 1). No subject scored within the normal range on the Introversion/Low Positive Emotionality (INTR/LPE) scale (Table 2). We distinguished and compared the low and high scorers on the INTR/LPE scale (Table 3). This study has limitations regarding sample size, higher-than-expected dropout rate, strict exclusion criteria, male-to-female ratio, short-term results, no longitudinal data on psychological characteristics.

**Image 1:**

**Figure:**
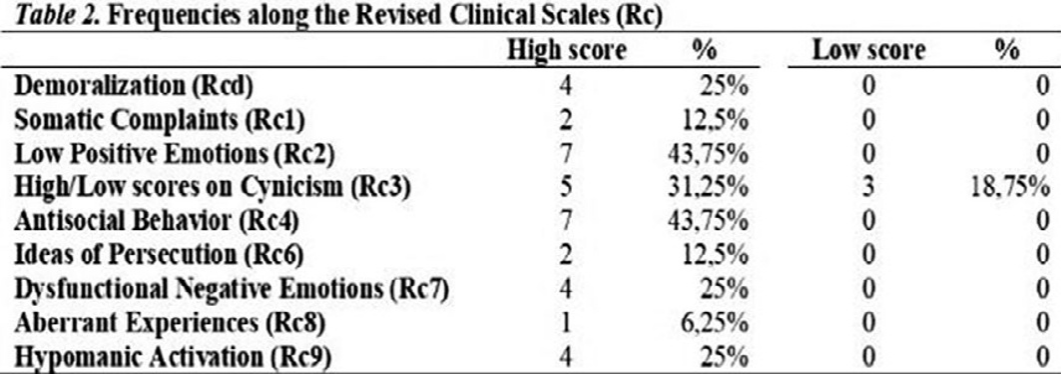

**Image 2:**

**Figure:**
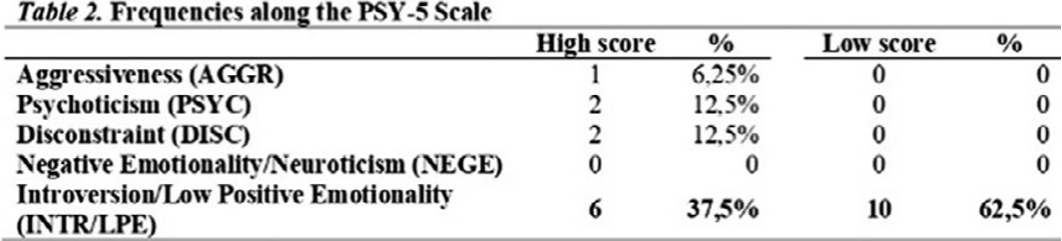

**Image 3:**

**Figure:**
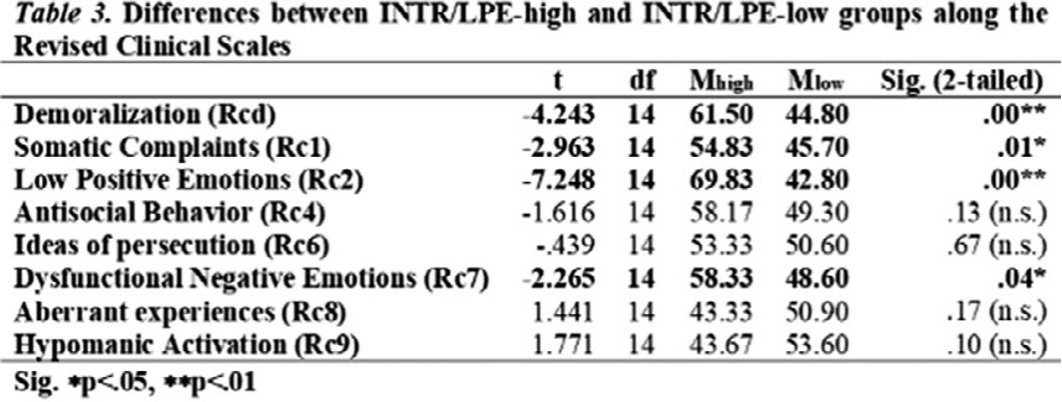

**Conclusions:**

Our results suggest that the majority of bariatric surgery patients, 12 months after the procedure, show signs of affective dysfunction, thought dysfunction and emotion dysregulation, all signs of a depressive state. Greater weight loss carries a greater probability of depression along with a lesser likelihood of positive emotional experiences; therefore, psychological support during follow-up is necessary to maintain weight loss.

**Disclosure of Interest:**

None Declared

